# Supplementary material for: The Application of Multi-Walled Carbon Nanotubes in Bone Tissue Repair Hybrid Scaffolds and the Effect on Cell Growth In Vitro
Source: Polymers (Basel). 2019 Feb 1;11(2):230. doi: 10.3390/polym11020230 (PMC6419040; doi:10.3390/polym11020230)
Supplement: Supplementary file 1 [file polymers-11-00230-s001.pdf]

Supplementary Materials: The Application of Multi-Walled Carbon Nanotubes in Bone Tissue Repair Hybrid Scaffolds and the Effect on Cell Growth In Vitro

Jie Xu, Xueyan Hu, Siyu Jiang, Yiwei Wang, Roxanne Parungao, Shuangshuang Zheng, Yi Nie *, Tianqing Liu * and Kedong Song

Table S1. Pore diameter data measured by Image-pro plus software of CS/Gel/nHAP scaffold and CS/Gel/nHAP/MWCNTs scaffolds. The average pore diameter of CS/Gel/nHAP, CS/Gel/nHAP/0.3%MWCNTs, CS/Gel/nHAP/0.6%MWCNTs and CS/Gel/nHAP/0.9%MWCNTs scaffold were about 176 μm, 178 μm, 169 μm and 160 μm, respectively.

Group	Control (μm)	0.3% (μm)	0.6% (μm)	0.9% (μm)
Dia.1	161.9471	279.8849	190.2872	162.5022
Dia.2	183.1502	256.4079	247.1572	188.9503
Dia.3	174.2129	161.5400	136.6163	135.2581
Dia.4	254.9032	175.6978	137.3965	160.5140
Dia.5	143.0242	145.3488	152.9939	136.9962
Dia.6	122.6716	145.3779	182.9518	131.6167
Dia.7	135.2512	123.6747	181.4677	140.2875
Dia.8	188.605	157.4068	154.5053	159.9867
Dia.9	137.1125	159.9894	175.5024	148.2818
Dia.10	173.7516	165.3658	156.4619	193.5237
Dia.11	198.7056	221.7320	172.7116	150.9365
Dia.12	146.0639	238.9563	228.9840	154.7512
Dia.13	191.0752	176.2740	155.4867	155.8124
Dia.14	118.3242	204.1103	226.5353	157.8401
Dia.15	197.5328	158.6900	227.0383	142.5284
Dia.16	168.0159	136.5041	158.6075	161.2231
Dia.17	219.3801	144.5618	157.6456	178.3221
Dia.18	179.9623	229.2092	127.9378	172.7116
Dia.19	176.8125	163.0241	119.6087	197.3716
Dia.20	241.4203	121.7118	93.2031	162.1638
Average	175.5961	178.2734	169.1549	159.5789

Note: Dia. is the abbreviation of diameter.

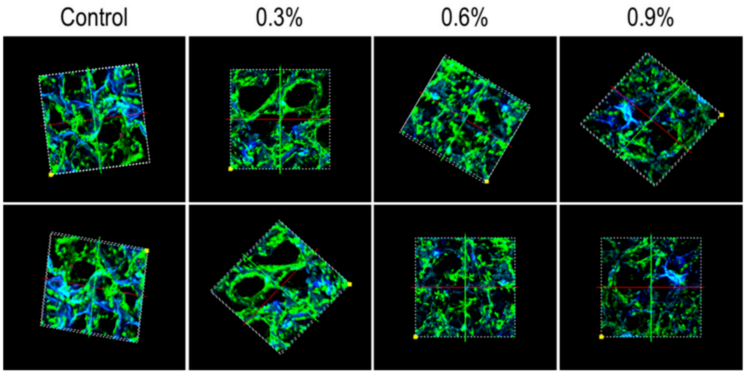

Figure S1. The 3D images distribution of MC3T3-E1 cells on different scaffolds. The 3D images show the 3D distribution observed from both sides of the scaffolds (due to the thin slice of 5 mm × 5 mm × 1 mm), and the MC3T3-E1 cells distributed on the different layers of the scaffolds.

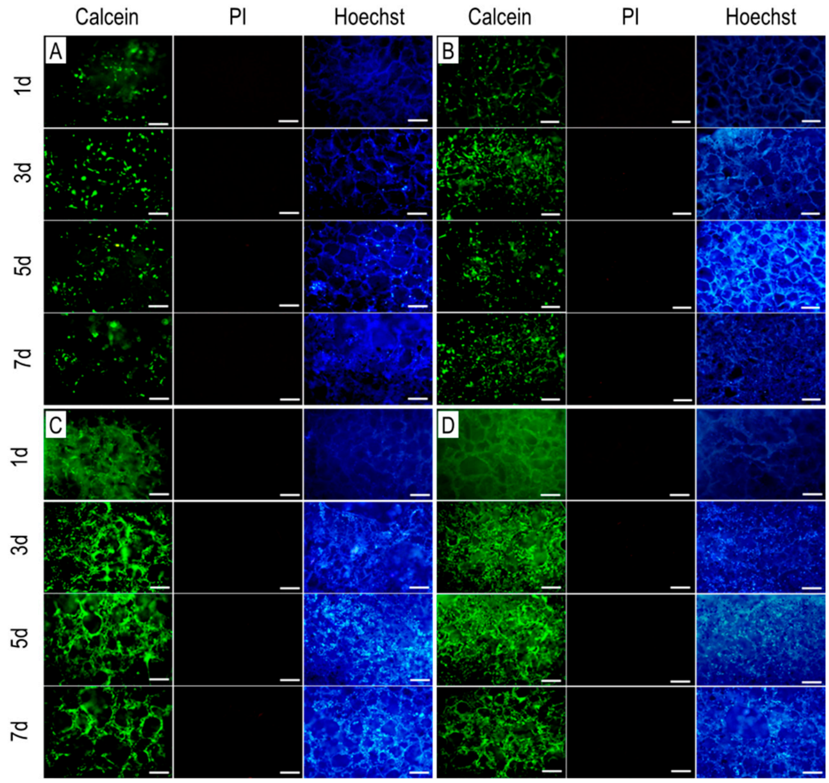

Figure S2. Viability and distribution of MC3T3-E1 cells on different scaffolds. The figure shows the live and dead cells cultured on different scaffolds for 1 day, 3 days, 5days and 7 days. (**A**) Control; (**B**) 0.3%; (**C**) 0.6%; (**D**) 0.9%; Scale: 250 μm.
